# First Report of *Dalbulus maidis* (DeLong and Wolcott) (Hemiptera: Cicadellidae) in Oklahoma

**DOI:** 10.3390/insects15100778

**Published:** 2024-10-08

**Authors:** Ashleigh M. Faris, Maira Rodrigues Duffeck, Jennifer D. Olson, Andres S. Espindola, Luana Muller, Sebastian E. Velasco, João Murilo Zambiasi

**Affiliations:** Department of Entomology and Plant Pathology, Oklahoma State University, Stillwater, OK 74078, USA; mairodr@okstate.edu (M.R.D.); jennifer.olson@okstate.edu (J.D.O.); andres.espindola@okstate.edu (A.S.E.); luana.muller@okstate.edu (L.M.); seespin@okstate.edu (S.E.V.); joao.zambiasi@okstate.edu (J.M.Z.)

**Keywords:** invasive insect, pest, leafhopper, maize, insect identification

## Abstract

**Simple Summary:**

The corn leafhopper, an invasive insect and vector of corn stunt spiroplasma, has been reported in multiple counties in Oklahoma. Corn stunt spiroplasma-infected corn leafhoppers were also confirmed in the state.

**Abstract:**

The corn leafhopper, *Dalbulus maidis* (DeLong and Wolcott) (Hemiptera: Cicadellidae), is an invasive insect that can cause damage to maize (*Zea mays* L.) in two ways: by direct feeding and the transmission of several plant pathogens. *Dalbulus maidis* is an invasive and serious economic pest of maize that has spread from its center of origin in Mexico to the southernmost parts of the United States. Prior to 2024, corn leafhoppers had not been documented in Oklahoma, and their spread northward toward the United States corn belt is of significant concern. Here, we provide the first reports of the insect in maize in several Oklahoma counties. Insect specimens were collected at various commercial and experimental field sites by Oklahoma State University research and extension personnel. The identity of the insect species was validated through morphological and molecular taxonomy. The presence records for the corn leafhopper presented here provide valuable information for future monitoring and management efforts of this economically important pest and disease.

## 1. Introduction

The corn leafhopper, *Dalbulus maidis* (DeLong and Wolcott) (Hemiptera: Cicadellidae), is an invasive insect that originates from Mexico, where it has evolved closely with modern maize (*Zea mays* L.) cultivars [1,2,3]. *Dalbulus maidis* is a serious economic pest of maize in Latin America due to the direct injury it causes to corn plants by feeding and its association with corn stunt disease [4,5,6,7,8]. The corn leafhopper has spread into parts of the southern United States, including California, New Mexico, Texas, Arkansas, Louisiana, Mississippi, and Florida, where in some locations sporadic outbreaks and economic loss have occurred [8,9,10,11]. While *D*. *maidis* is known to only reproduce in maize, studies have shown that when corn or teosintes are no longer available, the insect can survive in gamma grasses (*Tripsacum* spp.) [12], alfalfa (*Medicago sativa* L.), winter wheat (*Triticum aestivum* L.), triticale (*Triticale hexaploide* Lart.) [13], and possibly moist soil without plant material [3]. Studies also suggest that when *D. maidis* are infected as nymphs with *Spiroplasma kunkelii*, the causal agent for corn stunt disease [14], female *D. maidis* may have an improved overwintering survival [3,7,15].

In the United States, corn makes up more than 95% of feed grain production and use, with the majority produced in the heartland region [16]. Northern Oklahoma is located in the southernmost bounds of the United States heartland, and over the past three years, the state has increased corn acreage [17]. In 2023, Oklahoma planted 157,827 hectares of corn for grain and silage, with a USD 286,229,000 grain production value [17]. While Oklahoma corn production is overshadowed by states further north in the United States corn belt, the state’s annual acreage of winter wheat (over 1 million hectares in 2023) and alfalfa (70,819 hectares in 2023) may provide potential overwintering sites for adult *D*. *maidis* [17]. The arrival of *D*. *maidis* to the southern reaches of the heartland could have severe economic consequences for corn production should the insect become established in Oklahoma and continue to expand northward into the corn belt.

In August 2024, adult and nymph *D*. *maidis* were collected from several counties in Oklahoma. Specimen identification of adults based on morphological characters was determined by Ashleigh M. Faris (Oklahoma State University Department of Entomology and Plant Pathology) and confirmed by Astri Wayadande (Oklahoma State University). Molecular identification of *D*. *maidis* was confirmed by Sara Wallace (Oklahoma State University Plant Disease and Insect Diagnostic Laboratory (OSU PDIDL)). *S*. *kunkelii* was detected in adult *D*. *maidis* leg samples by the PDIDL. Here, we provide the first documented record of *D*. *maidis* in Oklahoma and discuss the concern for Oklahoma corn and other agricultural commodities to serve as a potential green bridge to the United States corn belt.

## 2. Materials and Methods

### 2.1. Collection of Dalbulus maidis

On 1 August 2024, Oklahoma State University Extension and Research personnel were notified of an insect infestation in a commercial corn field (35.401586, −96.765904) in Pottawatomie County, Oklahoma. *Dalbulus maidis* adults and nymphs were collected by hand and by using a sweep net from corn foliage. Following this initial collection, commercial and experimental corn field sites in West Central, Central, North Central, Panhandle, and Southwest districts were scouted for corn leafhoppers. The collected *D*. *maidis* were transferred to 50 mL Falcon^®^ (Corning Inc., Corning, NY, USA) tubes and stored at −80 °C.

### 2.2. Identification of Dalbulus maidis

Species identification was based on both morphological and molecular markers. The collected adult specimens were identified under a stereomicroscope at the OSU Cropping Systems Entomology Laboratory using the species-specific keys [18,19]. Male and female adult *D*. *maidis* were initially identified using external morphology. Abdomens from the male adult specimens were dissected from the insect, then cleared with 30% KOH for visualization of the genitalia, and identified for confirmation. Photographs of the male genitalia were obtained with an Olympus BX2 compound microscope equipped with a camera.

DNA was extracted at the OSU PDIDL from the body of the individual whole leafhoppers (*n* = 4) using the DNeasy^®^ Blood & Tissue Kit (Qiagen Inc., Hilden, Germany), per the manufacturer’s instructions [20]. The primers selected amplified a region of the mitochondrial cytochrome oxidase subunit I (*COI*) gene, previously used for *D*. *maidis* molecular identification [10,21,22]. The products were visualized on 1.5% agarose gel using gel electrophoresis with 1× TAE buffer under UV light and prepared for sequencing using the Gel extraction & PCR cleanup Kit (IBI Scientific, Dubuque, IA, USA). The purified DNA amplicon was sequenced from both ends using Sanger Dideoxi DNA sequencing at the OSU Biochemistry Core Facility. A consensus sequence was generated for each sample using Muscle 5.1 [23] and Geneious Prime^®^ 2024.0.7.

### 2.3. Molecular Confirmation of Dalbulus maidis Infected with Spiroplasma kunkelii

Detection of corn stunt spiroplasma, *S*. *kunkelii*, was attempted in *D*. *maidis* collected in corn fields from three Oklahoma counties. The legs from three adult corn leafhoppers were removed from the thorax using sterile forceps. Care was taken to ensure the forceps used to remove the legs did not puncture the insect. DNA was extracted from the pooled legs (*n* = 9) of three adult *D*. *maidis* (three legs from each adult, a combination of fore-, mid-, and hind legs) and from the pooled bodies from which the aforementioned legs were removed using the DNeasy^®^ Plant Mini Kit (Qiagen Inc., Hilden, Germany), per the manufacturer’s instructions [24]. The primers CSSF2 and CSSR6 were used to target a section of the *S*. *kunkelii* Spiralin gene [25]. The products were visualized on 1.5% agarose gel using gel electrophoresis with 1× TAE buffer under UV light and prepared for sequencing using the Gel extraction & PCR cleanup Kit (IBI Scientific, Dubuque, IA, USA). The purified PCR products were sequenced from both ends using the Sanger Dideoxy approach. A consensus sequence was generated for each sample using Muscle 5.1 [23] and Geneious Prime^®^ 2024.0.7.

## 3. Results

### 3.1. Collection of Dalbulus maidis

**Figure 1:**
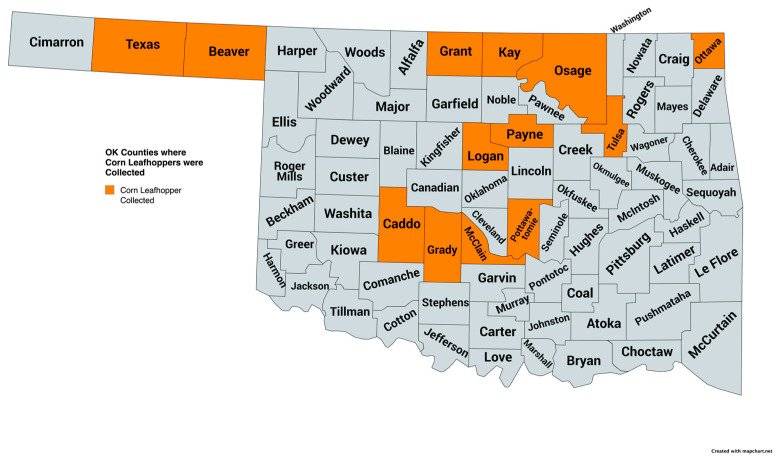
Oklahoma counties where corn leafhoppers, *Dalbulus maidis*, were collected from corn during August 2024. Map created by MapChart.net.

*Dalbulus maidis* were collected in thirteen Oklahoma counties during August 2024 (Figure 1). A minimum of three to five specimens were collected from a field during field visits as this work aimed to determine where *D*. *maidis* were present. All of the corn fields visited during August 2024 had *D*. *maidis* recovered. *Dalbulus maidis* were collected from irrigated and non-irrigated, commercial, and experimental field corn locations, as well as from commercial sweet corn (Table 1).

### 3.2. Identification of Dalbulus maidis

**Figure 2:**
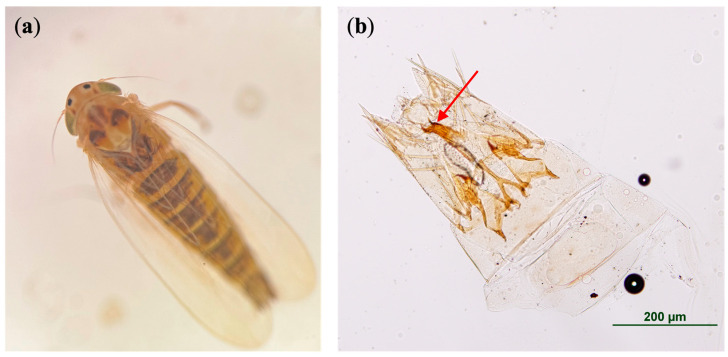
Microscope images of corn leafhopper. (**a**) The dorsal view of *Dalbulus maidis* adult. Note the two black spots between the eyes and the lack of prominent dark spots on the pronotum. (**b**) The ventral view of *D*. *maidis* male genitalia. The male aedeagus of this species is distinct due to two apical hooks (see the red arrow) and the lack of sclerotization of the ventral margin on the pygofer (last segment of the abdomen).

The specimens collected on maize in August 2024 were positively identified as *D*. *maidis* (Table 2). *Dalbulus maidis* adult specimens were first identified based on the presence of two dark spots between the eyes at the anterior margin of the head (Figure 2a) [18]. The identification of male adult *D*. *maidis* was confirmed by the presence of two distinct hooks on the distal end of the aedeagus and the ventral margin of the pygofer lacking sclerotization (Figure 2b) [18].

A BLASTn search of the consensus sequences of the COI gene indicated the identification of the four samples submitted to OSU PDIDL for molecular identification as *D*. *maidis* (Table 2), with a query coverage of 100% and a percent identity of 100%, with the accession number NC_070066.1. The sequenced fragments were deposited in GenBank with accession numbers PQ269316, PQ269317, PQ269318, and PQ269319.

### 3.3. Molecular Confirmation of Dalbulus maidis Infected with Spiroplasma kunkelii

A BLASTn search of the consensus sequences of the Spiralin gene indicated that the *D*. *maidis* leg and body samples from the initial Pottawatomie corn leafhopper outbreak tested positive for *S. kunkelii* (Table 3), with a query coverage of 100% and a percent identity of 100%, with the accession number KX925443.1. The sequenced fragments were deposited in GenBank with accession numbers PQ282393, PQ282394, PQ282395, and PQ282396. The leg and body samples for the other two locations were negative for *S*. *kunkelii* DNA (Table 3).

## 4. Discussion

Prior to the 2024 corn growing season, *D*. *maidis* had not been reported in Oklahoma. Communications by Texas A&M AgriLife Extension were distributed in late May of 2024 regarding a high number of *D*. *maidis* in the Texas Mid-Coast region. Before 2024, the most recent report of the insect south of Oklahoma was in 2016 from the lower Rio Grande Valley of Texas [10]. *Dalbulus maidis* outbreaks have been previously documented in United States corn production in California [8,9,13,26] and Florida [27,28], and the insect has been documented in Arizona, Louisiana, Arkansas, and Mississippi [29]. Due to the small size of the insect, prior observation of this insect in states that neighbor Oklahoma, and the ability of the insect to move through wind-aided movement, there is a possibility that the insect may have occurred in low, isolated numbers in Oklahoma before this first documented report. However, discussions with corn growers, crop consultants, entomologists, and industry personnel who have worked in Oklahoma corn production for the past forty-plus years indicate that this is the first growing season in which *D*. *maidis* was documented in the state (A.M.F, pers. obs.). At the time of the first collection of *D*. *maidis* in Pottawatomie County, located in central Oklahoma, the infestation of corn leafhoppers in that field was severe. The populations of *D*. *maidis* were not as high in the successive fields that were scouted for corn leafhoppers in August 2024. However, *D*. *maidis* were collected from every corn field scouted for this first detection work, including irrigated and non-irrigated fields. 

*Spiroplasma kunkelii*, one of the pathogens associated with corn stunt disease, was detected in the bodies and legs of the insects from the initial field site. Detection in the body indicates that the insect fed on an *S*. *kunkelii*-infected plants. Phytopathogenic spiroplasmas, including *S. kunkelii*, are propagative bacteria and must cross both the gut and salivary gland barriers within the body of their vector before they can be inoculated (or transmitted) into a plant host [30]. Detection of the pathogen in the legs of the insect indicates that the insect was likely to be systemically infected and had the ability to transmit the corn stunt spiroplasma [1,31]. For the leg and body samples that were tested for *S*. *kunkelii* and yielded a negative result, it is possible that the spiroplasma was in the latent period (18–22 days) and not yet acquired systemically [1]. At the time of the collections in the summer of 2024, there were *D*. *maidis* capable of transmitting *S*. *kunkelii* in Oklahoma corn production.

Corn is planted at different times in Oklahoma, with early planting in mid- to late March, full-season planting in late March to early April, and double-crop planting in July, creating an environment where *D. maidis* can find suitable reproductive material for several months during the corn growing season. Double-crop corn planting follows winter wheat harvest in Oklahoma, allowing growers to plant and harvest two crops in a single year. Early-planted and full-season corn is generally harvested in late July through August, and double-crop corn is harvested in September. Once the corn is harvested, growers will plant winter wheat in the corn fields, leading to the opportunity for volunteer corn. There are also several homeowners and market farmers who grow sweet corn in succession in Oklahoma to have sweet corn available for food or sale throughout the summer and fall months. In terms of scale, these operations are often smaller than commercial field corn operations. Still, the proximity of successive sweet corn operations to field corn acres may also be a resource for *D. maidis* over an extended period. Based on previous studies, *D*. *maidis* can survive without corn for up to 9 weeks so long as water is available [12].

Studies have shown that *D*. *maidis* will overwinter as adults. When pre-conditioned to cooler temperatures, *D*. *maidis* has an improved overwintering survivability, surviving at temperatures as low as −5 °C for just over 8 h [13,32] and for several days at ≈5 °C [13]. Typically, the first winter frost in Oklahoma will occur in late October for the northern counties and early to mid-November for the central to southern counties [33]. But with warming temperatures, the first frost date has shifted to early to mid-November for much of the state. Due to a mild winter with warm spring temperatures, there were reports of corn planted as early as late February in 2024 in Oklahoma. The ability of *D*. *maidis* to fly long distances [34] aided by wind, along with warming temperatures which allow for earlier planting, may be why the insect is now observed in Oklahoma.

The impact that corn leafhopper infestations may have on corn yield in 2024 is unclear due to the timing of the insect being detected late in the season for early-planted and full-season corn. Efforts are ongoing to monitor *D*. *maidis* in double-crop and late-planted corn, as well as monitoring potential overwintering sites for the insect in Oklahoma. Due to the potential for earlier planting and volunteer corn following harvest, there are locations in Oklahoma where corn may be an available resource for nine months. Depending on environmental conditions, *D*. *maidis* may be able to reproduce on volunteer corn and potentially move to overwintering sites once the volunteer corn dies at first frost [13]. Identifying potential *D*. *maidis* overwintering sites in Oklahoma may play an important role in identifying ways to manage insect populations should *D*. *maidis* overwinter in the state. If it is confirmed that the insect can overwinter in Oklahoma, corn production may be at risk of economic loss. The impact may also be felt in the beef cattle production sector, the number one agriculture industry in Oklahoma (USD 6.06 billion in 2022), due to losses of corn for silage and feed [35].

## 5. Conclusions

The first detection of *D*. *maidis* in Oklahoma—and its widespread distribution across the state—is a concern for corn growers in the state and their northern neighbors in the United States corn belt. If conditions persist that are conducive to *D. maidis* populations reproducing and overwintering in the state, then not only would Oklahoma corn production be at risk of economic losses, but so may the major grain-producing region of the United States and the world. In addition to the prolonged corn growing season in Oklahoma and the potential for adult overwintering conditions and sites, additional challenges exist. A recent study in Brazil has shown *D*. *maidis* to have high susceptibility to the insecticides methomyl, carbosulfan, and acephate [36]. Unfortunately, in the United States, these insecticides may not be available for pest management like they are in other countries. Carbosulfan is not registered for use in the United States. Although acephate is a commonly used insecticide for agronomic pests in the United States, it is not registered for use in corn. Additionally, methomyl is currently under scrutiny from the Environmental Protection Agency due to health concerns for endangered species and humans [37]. Pyrethroids are a common insecticide that growers prefer to use due to their general low cost. However, the aforementioned study showed that *D*. *maidis* had reduced susceptibility to both bifenthrin (pyrethroid) and imidacloprid (neonicotinoid) [36], which is a commonly used insecticidal seed treatment in United States corn production. Oklahoma and other states may face challenges not previously experienced in countries where *D*. *maidis* is endemic.

Future work on *D*. *maidis* in Oklahoma will consist of continued monitoring for the insect both in and out of the corn growing season across the state. Presence and absence data will be beneficial for identifying potential overwintering sites that may need targeted management practices for *D*. *maidis* populations. Determining when, where, and at what temperature *D*. *maidis* is last detected in the state will also be useful for developing management practices if needed after the Oklahoma winter season. Future work will also assess the impact of *D*. *maidis* on Oklahoma corn yield.

## Figures and Tables

**Table 1:** Irrigation practice and corn type by county for fields where *Dalbulus maidis* were collected in August 2024.

County	Field Type	Irrigated	Corn Type
Beaver	Commercial	Yes	Field Corn
Caddo	Commercial	Yes	Field Corn
Grady	Commercial	Yes	Field Corn
Grant	Commercial	No	Field Corn
Kay	Commercial	No	Field Corn
Logan	Commercial	Yes	Sweet Corn
McClain	Commercial	Yes	Field Corn
Osage	Commercial	No	Field Corn
Ottawa	Commercial	No	Field Corn
Payne	Experimental	Yes and No	Field Corn
Pottawatomie	Commercial	Yes	Field Corn
Texas	Commercial and Experimental	Yes	Field Corn
Tulsa	Experimental	Yes	Field Corn

**Table 2:** Georeferenced locations for adult samples confirmed to be *Dalbulus maidis*. All specimens were collected from corn foliage.

County Location	Latitude	Longitude	Identification *
Beaver	36.620775	−100.773839	Morph.
Caddo	35.579137	−98.5850329	Morph.
	35.446107	−98.575681	Morph.
	35.230304	−98.4072021	Morph.
	35.2151539	−98.414483	Morph.
Grady	35.03199	−97.899806	Morph.
	35.033779	−97.811804	Morph.
Grant	36.5960894	−97.8723234	Morph.
Kay	36.7239736	−97.2282522	Morph.
	36.7407845	−97.2181628	Morph.
Logan	35.959208	−97.190875	Morph.
McClain	35.228715	−97.599452	Morph.
Osage	36.9337746	−96.9420521	Morph.
	36.9498224	−96.9382203	Morph.
Ottawa	36.9291089	−94.818898	Morph. and Molec.
	36.8030767	−94.8931194	Morph. and Molec.
Payne	36.133201	−97.106187	Morph.
	36.133169	−97.105644	Morph.
Pottawatomie	35.399688	−96.768343	Morph.
Texas	36.659166	−101.42933	Morph. and Molec.
Tulsa	35.9652956	−95.8605271	Morph.
Tulsa	35.964379	−95.865886	Morph. and Molec.

* Morph.: morphological identification; Molec.: molecular identification.

**Table 3:** Field sites where *S*. *kunkelii* was molecularly confirmed in *D*. *maidis* legs and bodies. “(+)” indicates positive for *S*. *kunkelii*; “(−)” indicates negative for *S*. *kunkelii*.

County Location	Latitude	Longitude	*S*. *kunkelii* Confirmation
Pottawatomie	35.399688	−96.768343	(+) Legs, (+) Bodies
Grady	35.03199	−97.899806	(−) Legs, (−) Bodies
Payne	36.133169	−97.105644	(−) Legs, (−) Bodies

## Data Availability

The raw data supporting the conclusions of this article will be made available by the authors on request.
